# The Relationship Between Gratitude and Loneliness: The Potential Benefits of Gratitude for Promoting Social Bonds

**DOI:** 10.5964/ejop.v11i2.826

**Published:** 2015-05-29

**Authors:** Andrea Caputo

**Affiliations:** a Department of Dynamic and Clinical Psychology, University of Rome “Sapienza”, Rome, Italy; Academy of Special Education, Warsaw, Poland

**Keywords:** gratitude, loneliness, social relationships, social desirability, well-being

## Abstract

This paper explores the potential role of gratitude on the reduction of loneliness feelings, even controlling for several variables related to social desirability, well-being (subjective happiness and life satisfaction) and socio-demographic characteristics. Through a web-based survey a convenience sample of 197 participants completed an online questionnaire including these measures. Correlation analyses and four-step hierarchical multiple regression analyses were conducted. The results show a negative correlation between gratitude and loneliness; specifically, gratitude succeeds in accounting for up to almost one-fifth of the total variability of loneliness even controlling for further variables. Being female, not having a stable and consolidated relationship and not participating in the labor force represent some risk factors affecting loneliness which should be taken into account in further research.

Loneliness plays an important role in investigating the psychological process of human feelings and behaviors with regard to the formation and maintenance of social relationships (Wu & Yao, 2008). Loneliness is an emotionally unpleasant experience resulting from a discrepancy between the types of interpersonal relationships one wishes to have, and those that one perceives they presently have (Peplau & Perlman, 1982). It can be considered as a feeling of emptiness, isolation or unwanted solitude, clearly distinguishable from the objective state of solitude, social isolation, or being alone. Indeed, social contact does not necessarily buffer one against loneliness because the experience of loneliness seems to have more to do with individuals’ perceptions of the quality of social interactions (Asher & Paquette, 2003; Hawkley, Burleson, Berntson, & Cacioppo, 2003).

Many studies (Hawkley & Cacioppo, 2010; Heinrich & Gullone, 2006) indicated that loneliness can negatively affect health, life satisfaction and well-being. In this regard, most of the recent research on subjective well-being has focused on the benefits of gratitude as a means to increase life satisfaction and positive affects and decrease negative ones from childhood to old age (Emmons & McCullough, 2003; Watkins, Woodward, Stone, & Kolts, 2003). Gratitude is recognized as “indispensable in the life of one individual who will face isolation and loneliness if the capacity to feel grateful is impaired” (Emmons & McCullough, 2004, p. 2010), because it motivates the reciprocation of aid (Bartlett & DeSteno, 2006; McCullough, Kilpatrick, Emmons, & Larson, 2001; Tsang, 2006). As stated by Melanie Klein (1963/1975) gratitude can mitigate loneliness because it deeply includes a very close connection between being able to accept and to give, and both are part of the relation with the good object. Indeed, loneliness relates to a deficiency of the needs of intimacy and meaning (McGraw, 1995). Expressing gratitude thus plays an important role in relationships because it can strengthen social bonds and friendships (Emmons & Shelton, 2002; McCullough & Tsang, 2004), as well as the characteristics needed for their development and maintenance (Algoe, Haidt, & Gable, 2008; Gordon, Arnette, & Smith, 2011; Kubacka, Finkenauer, Rusbult, & Keijsers, 2011; McCullough, Kimeldorf, & Cohen, 2008), such as peer, family and social support (Froh, Yurkewicz, & Kashdan, 2009), prosocial motivation (Michie, 2009; Naito, Wangwan, & Tani, 2005; Tsang, 2006), relationship connection and satisfaction (Algoe, Gable, & Maisel, 2010), willingness to forgive (DeShea, 2003), praise (Deutsch, Roksa, & Meeske, 2003) and trust (Dunn & Schweitzer, 2005). However, only few studies have specifically examined the potential role of gratitude for the reduction of loneliness feelings (Burcat, 2010; Feng, 2011). In addition, these studies show two main limitations: the specific type of samples selected (respectively, college students at a public California university in the study by Burcat and Korean American pastors and spouses in the study by Feng) and the correlation analyses conducted which did not take into account further confounding factors.

Therefore, this paper aims at proposing a research study which consents to overcome these two limitations by extending findings to a wider range of population and using regression models to disentangle the relationship between gratitude and loneliness controlling for further variables.

## Theoretical Framework and Aim of the Study

In this study loneliness is defined as an individual’s subjective experience of emptiness, isolation and lack of satisfying human relationships (Hughes, Waite, Hawkley, & Cacioppo, 2004; Victor, Scambler, Bond, & Bowling, 2000), thus causing negative feelings and distress. Loneliness “denotes the lack of intimate/meaningful solidarity with other beings and bespeaks an entitative-emotional longing for their plenitude and connectedness” (McGraw, 1995, p. 46). In this sense, gratitude is hypothesized to reduce the feeling of individual isolation from others resulting in insecurity and instability (McGraw, 1995), which impacts on how people interact, as well as how they interpret interpersonal situations (Murphy & Kupshik, 1992). The emotion of gratitude can influence the likelihood of their forming satisfying relationships, counteracting the distorted thinking which can lead to loneliness by causing deficits in sociability (Peplau & Perlman, 1982). By distorted thinking we mean irrational cognitions according to which, in explaining the causes of their loneliness, lonely people are likely to blame themselves, deriving uncontrollable, internal, and stable attributions (Solano, 1987). That is, they are likely to see their personalities as unchangeable (stable), view social situations as being beyond their control, and believe that they do not have friends because they are perhaps dull and boring (internal). This contributes to inappropriate patterns of self-disclosure (Horowitz & French, 1979), less effective coping behavior and dysfunctional attitudes (e.g., fears of interpersonal rejection, feeling unsure of oneself, and social anxiety) (Wilbert & Rupert, 1986). Instead, grateful individuals can have an expanded circle of attributions, because they attribute their success to others’ controllable actions (Weiner, 1985), and also take into account how they themselves have contributed to their own success (McCullough, Emmons, & Tsang, 2002). Therefore, the experience of gratitude may allow people to be more responsive to others and take full advantage of available interpersonal opportunities (Jones, Hobbs, & Hockenbury, 1982), thus increasing self-perceived social competence and interrupting chronic loneliness which impede the future meeting of belongingness needs.

This study aims at investigating the potential role of gratitude on the reduction of loneliness feelings. In doing that, it takes into account some confounding factors which could interfere in this relationship, which mainly refer to social desirability bias and other positive emotions. On the one hand, social desirability may affect both gratitude and loneliness because it leads to report pro-social and altruistic dispositions which are culturally agreeable (Lyubomirsky, Sheldon, & Schkade, 2005; Wood, Froh, & Geraghty, 2010), as well as to hide undesirable feelings related to low emotional well-being (Lasgaard, Goossens, & Elklit, 2011). On the other hand, how much of the variance in loneliness is due to gratitude needs to be studied independently of how much it is due to well-being measures such as life satisfaction and subjective happiness.

## Method

### Participants and Procedure

A web-based survey was promoted via social media (forums, blogs, social networks) to study the relationship between subjective well-being and other related psychological constructs. It was conducted according to online survey design, development and implementation guidelines suggested by Andrews, Nonnecke, and Preece (2003). Online survey was chosen because of its widespread use for quality of life, health-related and well-being research (Vereecken & Maes, 2006) and its easy access to geographically diverse respondent groups across the national context (Evans & Mathur, 2005). In addition, the validity and reliability of internet research for subjective well-being surveys were demonstrated to be comparable to those of the paper-based versions (Howell, Rodzon, Kurai, & Sanchez, 2010).

A convenience sample of 197 participants was recruited (158 women and 39 men) whose mean age was 29.1 (*SD* = 10.4). A questionnaire was administered which included socio-demographic information and loneliness, gratitude, social desirability, subjective happiness and life satisfaction measures. Participants were guaranteed anonymity. For the present study 100% of the respondents filled in the complete questionnaire without missing data.

### Measures

#### Gratitude

The *Gratitude Questionnaire-Six-Item Form* (GQ-6) (McCullough, Emmons, & Tsang, 2002) is a six- item self-report questionnaire designed to assess individual differences in the proneness to experience expressions of gratefulness and appreciation in daily life, as well as feelings about receiving from others. Respondents endorsed each item on a 7-point Likert-type scale and the score was calculated as the sum of items, ranging from 6 to 42. Higher scores mean higher proneness to experience gratitude in daily life. For the purpose of this study, the scale was adapted to the Italian language through translation, back translation and equivalence evaluation, and showed a satisfactory internal consistency (α = .750).

#### Loneliness

The *Three-Item Loneliness Scale*, developed by Hughes et al. (2004) from the revised *UCLA Loneliness Scale*, was used to assess loneliness consisting of feelings of isolation, disconnectedness, and not belonging. The response categories were coded 1 (hardly ever), 2 (some of the time), and 3 (often) on a 3-point scale. Each person’s responses to the questions are summed, with higher scores indicating greater loneliness. For the purpose of this study, the three items were derived from the Italian version of the revised *UCLA Loneliness Scale* (Solano & Coda, 1994). The 3-item scale showed a good internal consistency (α = .839).

#### Social desirability

The Italian adaptation (Manganelli Rattazzi, Canova, & Marcorin, 2000) of the short 9-item version of the *Marlowe-Crowne Social Desirability Scale* (MC-SDS) was used to measure social desirability. Participants were requested to respond to each item on a 7-point scale. A total score is derived from the sum of all items, ranging from 7 to 63. Higher scores indicate higher levels of social desirability. Internal consistency was sufficient (α = .611). The relatively low Cronbach’s alpha seems to be in agreement with other studies using the Italian short version of the MC-SDS (Maino & Aceti, 1997; Manganelli Rattazzi et al., 2000).

#### Subjective happiness

The *Subjective Happiness Scale* (SHS) (Lyubomirsky & Lepper, 1999) is a widely used four-item scale, measuring global subjective happiness. The scale required participants to use absolute ratings to characterize themselves as happy or unhappy individuals on a 7-point Likert scale, as well as it asked to what extent they identify themselves with description of happy and unhappy individuals. The score was calculated as the mean of items, ranging from 1 to 7. Higher scores mean greater perceived happiness. The Italian version of the scale was used (Duncan & Grazzani-Gavazzi, 2004) and showed a Cronbach’s alpha of .847.

#### Life satisfaction

As a measure of global life satisfaction, a three-item scale was specifically developed and used for the purpose of the study. Subjects had to rate how much they were satisfied with three dimensions respectively regarding socio-economic status, general health status, and life style and conditions, using a 10-point agreement scale. The score was calculated as the sum of items, ranging from 3 to 30. Higher scores mean better life satisfaction. The scale showed good psychometric properties with a Cronbach’s alpha of .764.

### Statistical Procedures

To explore the relationships among the different measures considered (gratitude, loneliness, social desirability, subjective happiness, life satisfaction), correlation analyses were performed.

With regard to our research question, four-step hierarchical multiple regression analyses were conducted by using loneliness measure as dependent variable. The Model 1 includes only gratitude measure as explicative variable in order to test whether gratitude can explain variance of loneliness. Then other variables were progressively entered in the regression models in order to estimate the predictive value of gratitude in reducing loneliness, even controlling for several additional characteristics which refer to: social desirability bias (Model 2), well-being measures such as subjective happiness and life satisfaction (Model 3) and socio-demographic variables (Model 4). With regard to socio-demographic predictors, a dummy for gender (male, female) and three dummies respectively for marital/relationship status (married/cohabitant, in a relationship, single) and employment status (employed, unemployed, others) were created. Instead, age and education were inserted as continuous variables. All analyses were performed using SPSS 16.0.

## Results

In Table 1 socio-demographic variables of our sample are reported, as well as descriptive characteristics of used measures.

**Table 1:** Socio-Demographic Variables and Descriptive Characteristics of Subjective Well-Being Measures of the Sample (N = 197)

Socio-demographic variables			Subjective Happiness		Life Satisfaction		Loneliness		Gratitude		Social Desirability	
Categorical variables	*n*	*%*	*M*	*SD*	*M*	*SD*	*M*	*SD*	*M*	*SD*	*M*	*SD*
*Gender*												
Male	39	19.8	4.01	1.16	18.97	4.76	4.97	1.80	(6.92)	24.59	38.64	7.22
Female	158	80.2	3.87	1.04	18.54	5.16	5.73	2.09	25.54	7.41	37.59	7.66
*Marital/Relationship status*												
Married-Cohabitant	56	28.4	4.22	1.04	19.48	4.97	4.54	1.78	27.70	7.40	39.20	8.20
In a relationship	62	31.5	3.89	0.93	18.90	4.56	5.74	1.98	24.08	6.35	37.21	6.79
Single	79	40.1	3.68	1.13	17.81	5.46	6.20	2.02	25.14	7.79	37.28	7.65
*Employment status*												
Employed	85	43.1	4.08	0.95	19.25	4.78	5.09	1.86	26.51	6.99	37.20	7.86
Unemployed	33	16.8	3.48	1.07	15.79	5.54	5.79	2.23	21.73	7.05	36.18	7.67
Others^a^	79	40.1	3.88	1.13	19.15	4.85	6.03	2.08	25.63	7.07	36.16	7.08
Continuous variables	*M*	*SD*										
*Age*	29.10	10.40	-	-	-		-				-	
*Education (years)*	13.45	3.52	-	-	-		-				-	
Total			3.90	1.06	18.63	5.08	5.58	2.05	25.36	7.31	37.80	7.57

^a^Others include homemakers, students or retired.

As shown in Table 2, the negative correlation between loneliness and gratitude is confirmed and the other measures are associated accordingly with the theoretical framework: loneliness is negatively correlated with subjective happiness, life satisfaction and social desirability; while gratitude shows a positive association with them. However, the correlations are not so high to cause potential multicollinearity problems in regression analyses, thus indicating the presence of separate constructs.

**Table 2:** Correlations for all Measures

	Subjective Happiness	Life satisfaction	Loneliness	Gratitude	Social Desirability
Subjective Happiness	1				
Life satisfaction	.601***	1			
Loneliness	-.557***	-.481***	1		
Gratitude	.483***	.463***	-.438***	1	
Social Desirability	.168**	.235***	-.253***	.316***	1

**p < .01. ***p < .001.

The results of hierarchical multiple regression analyses (Table 3) indicate that gratitude can be considered as a valid predictor in all the regression models, accounting for up to 18.7% of the total variance of loneliness. Gratitude thus seems to contribute to reduce loneliness feelings. When entering additional controls, its coefficient tends to decrease because of its relationship with the other explicative variables, despite it still remains statistically significant. In the Model 2, social desirability does not show an incremental validity in explaining loneliness. Instead, in the Model 3, the well-being controls increase the overall explained variance thus suggesting the relevance of individual positive emotional states in accounting loneliness. Then, in the Model 4 (explaining 44.4% of loneliness) the results about socio-demographic characteristics show that being male and married or cohabitant predict lower loneliness, while homemakers, students or retired (in terms of occupational status) generally feel more lonely. In other words, this means that being female, not having a stable and consolidated relationship and not participating in the labor force represent some risk factors affecting loneliness which should be taken into account.

**Table 3:** Summary of Hierarchical Multiple Regression Analyses for Predicting Loneliness (Standardized Coefficients)

	Model 1	Model 2	Model 3	Model 4
Constant	8.699***	9.722***	11.747***	12.051***
Gratitude	-0.438***	-0.397***	-0.150*	-0.176**
Social desirability		-0.128	-0.105	-0.054
Subjective happiness			-0.367***	-0.312***
Life satisfaction			-0.166*	-0.186*
Gender (Male)				-0.122*
Age				-0.087
Education				-0.063
Marital/Relationship status (ref: Single)				
Married-Cohabitant				-0.201**
In a relationship				-0.082
Employment status (ref: Unemployed)				
Employed				0.143
Others				0.186*
**R**	0.438	0.454	0.614	0.689
**R^2^ (Adjusted)**	0.187***	0.198***	0.364***	0.444***
**∆R^2^**	0.192***	0.015	0.171***	0.098**

*p < .05. **p < .01. ***p < .001.

## Discussion

The study results highlight that loneliness and gratitude are negatively correlated, consistently with previous research (Burcat, 2010; Feng, 2011). In addition, gratitude is demonstrated to be associated with increase in happiness, life satisfaction and social desirability; differently from loneliness which is generally related to negative outcomes in these measures. With regard to our research aim, gratitude seems to work as an influential moderator of loneliness feelings, accounting for up to almost one-fifth of its variability, thus suggesting the potential benefits of gratitude for promoting social bonds.

Some gender differences in predicting loneliness emerge which indicate that women are more vulnerable to loneliness than men (Brackin, 2002; Briscoe, 2005; Victor, Scambler, Bowling, & Bond, 2005). Gilligan (1982) suggested that women may develop a way of thinking about the world that depends on a sense of being connected with others. Thus, women feel that to be alone is to be a failure. Men, by contrast, value independence, and believe that it is unmanly to need another to assuage loneliness. However, previous research findings have been quite ambiguous and controversial in this regard (Mahon, Yarcheski, Yarcheski, Cannella, & Hanks, 2006). Borys and Perlman (1985) found differentiated results of gender differences in loneliness using different measures. In detail, they highlighted that when loneliness is measured using the direct self-labeling measurement (e.g., “do you often feel lonely?”) as in our study, females reported higher level of loneliness.

Besides, higher levels of loneliness are perceived in people who are single than in married/cohabitant ones, consistently with recent literature (Drennan et al., 2008; Ernst & Cacioppo, 1999; Findlay, 2003; Victor et al., 2005). Indeed, data from respondents in more than 20 nations document that loneliness is less common among married than non-married individuals (Perlman & Peplau, 1998). Being married is considered as a protective factor in the experience of loneliness (Lauder, Sharkey, & Mummery, 2004), probably because of the socially integrating function of a stable partner (Dykstra, 1995). The presence of a partner facilitates social interaction and many activities are undertaken as a couple, with other couples; while the absence of an important relationship or the lost connection with a loved one can lead to loneliness (Mcinnis, 2000).

Then, loneliness mainly characterizes homemakers, students or retired than other social categories probably because they do not participate in the labor force and may have more restricted social networks. Indeed, college students are more lonely than older adults (Rokach, 2000), homemakers may build fewer nonfamily social contacts due to the lack of opportunities to build such contacts through their employment (Owens & Swensen, 2000), and retired people may be characterized by reduced mobility as a precondition for seeking out contact partners (Dawson, Hendershot, & Fulton, 1987) and facilitating the establishment and maintenance of social contacts. This notwithstanding, the comparison between these categories and unemployed people (who don’t report higher loneliness in our study) should be further examined in future research in order to better understand the specific factors explaining these differences.

Some limitations regarding this study need to be taken into account in order to put the findings into perspective. At first, this study used a convenience sample composed of internet users which was not a national representation and was not randomly chosen. Issues regarding self-selection bias may thus exist which do not allow generalization. In addition, internet users may be characterized by increased loneliness and a reduction in both the number of friends people have and the time they spend with them (Kraut et al., 1998). However, other studies found no such correlations (Katz & Aspden, 1997) or achieved opposite conclusions (Coget, Yamauchi, & Suman, 2002). Another limitation refers to the low generalizability of findings due to the small number of male respondents in the sample. However, the inclusion of gender as covariate in the regression analysis helped adjust estimates for this gender imbalance. Then, the nature of this research does not enable conclusions on causal relations between examined variables, because correlation analyses and self-report measures also have inherent limitations. Another limitation concerns the causal relationship between gratitude and loneliness. In this regard, loneliness might also be considered as a moderating factor that can facilitate (or not) the opportunity to build social bond and the consequent feeling of gratitude. However, longitudinal data would be required to disentangle the pattern of these causal effects. In addition, experimental research could be carried out where participants could be induced to engage in various amounts of gratitude to examine what effects this would have on their feelings of loneliness.
